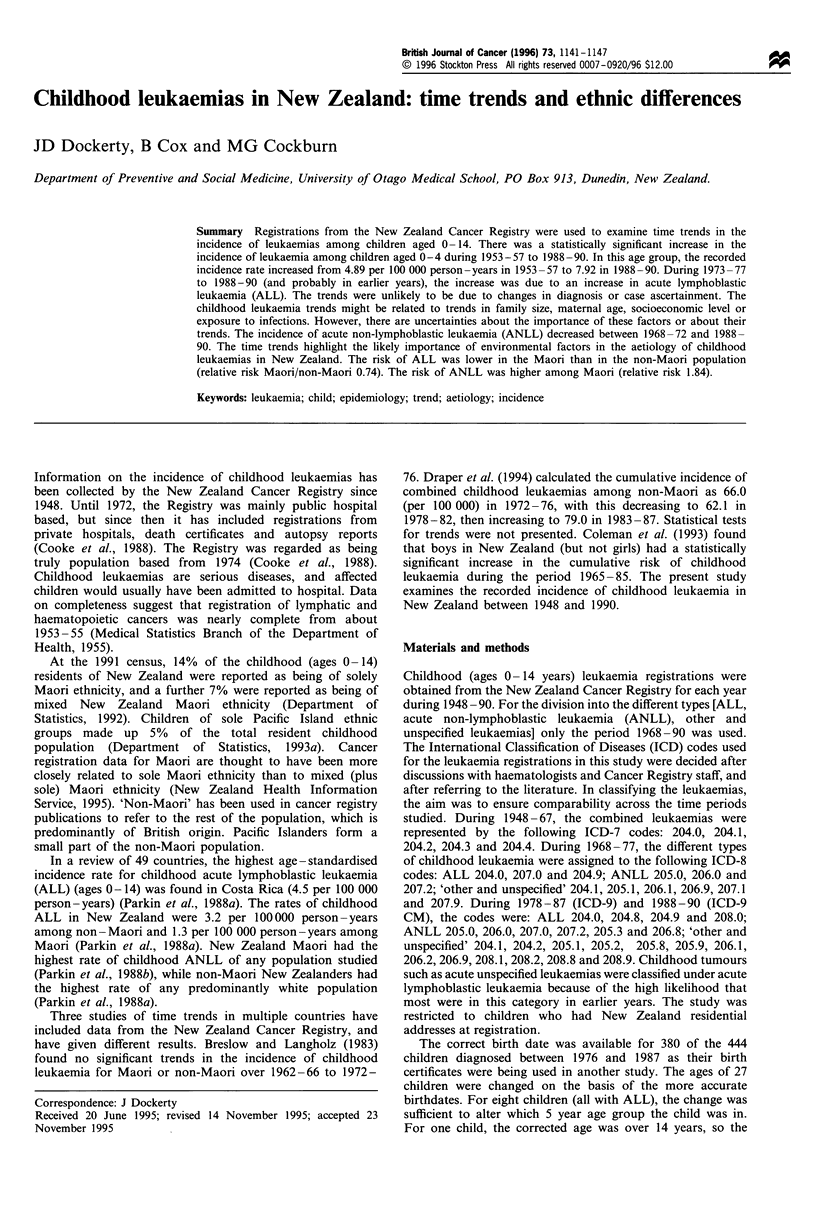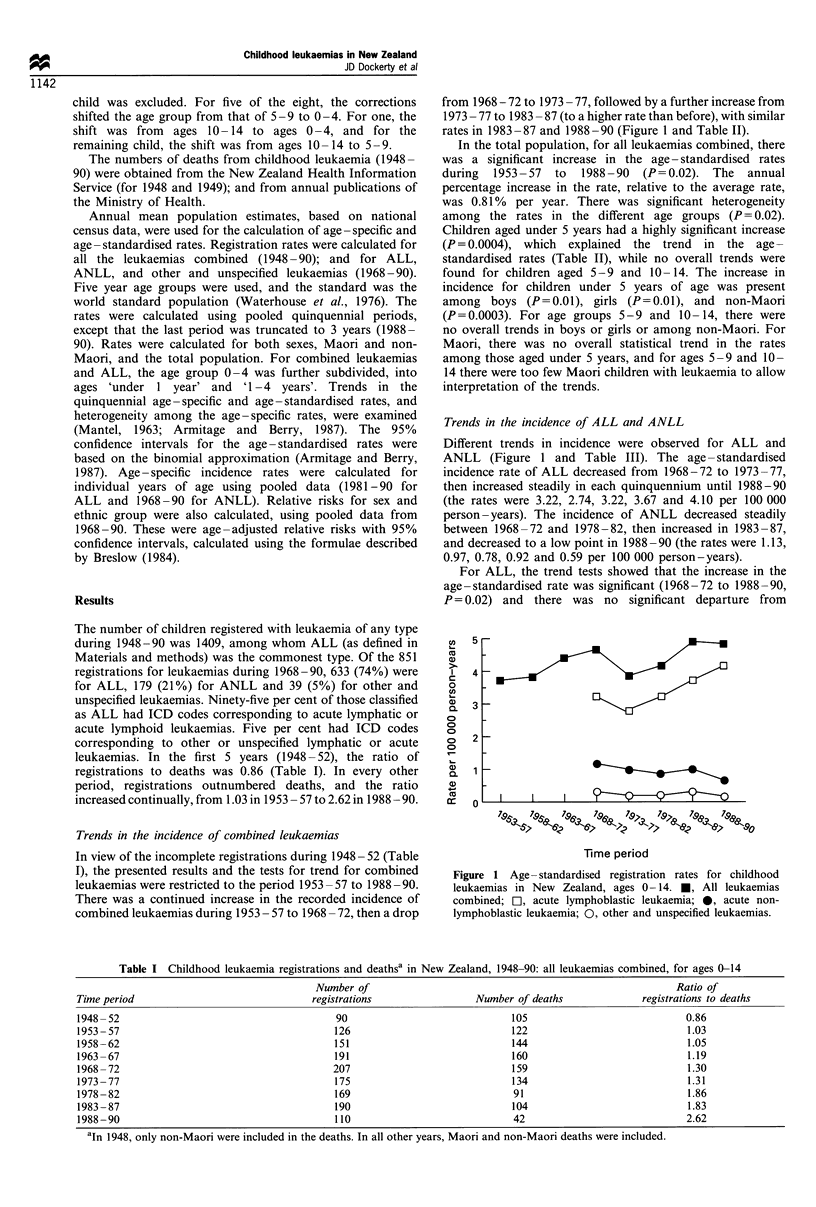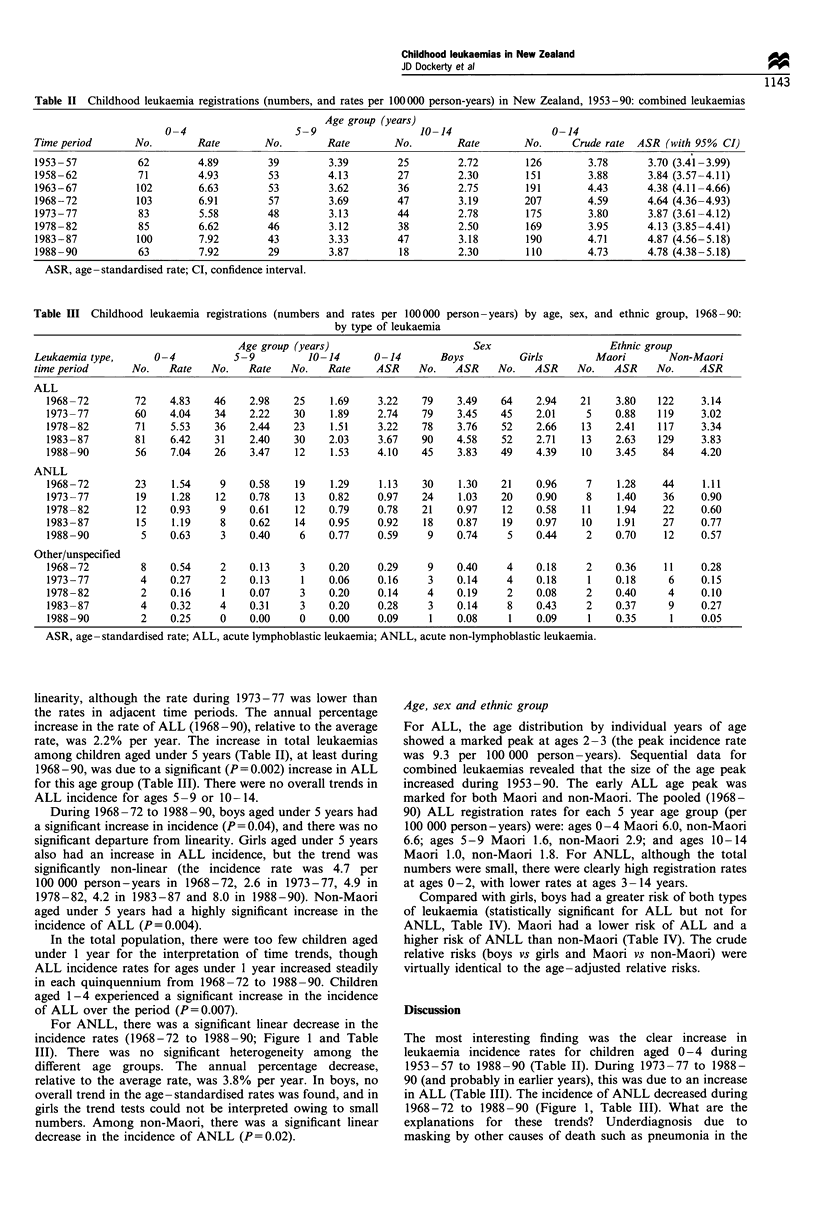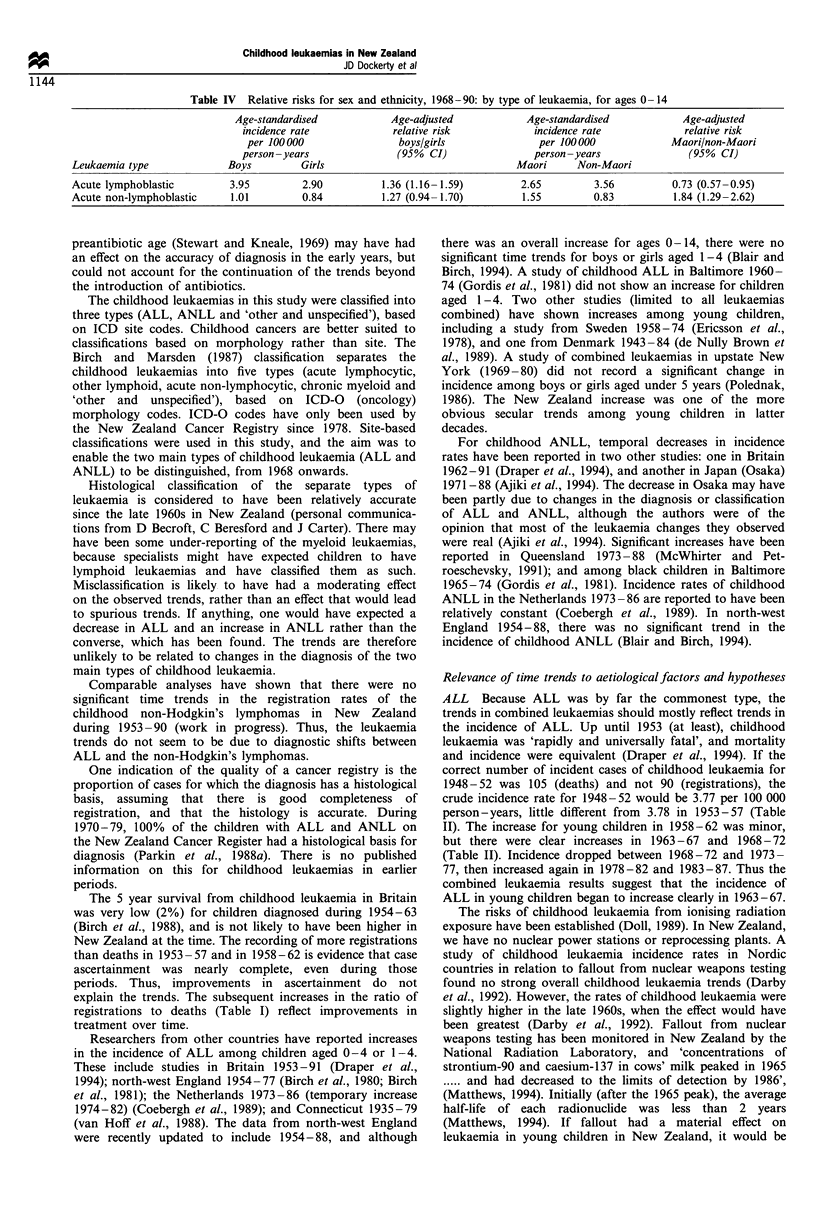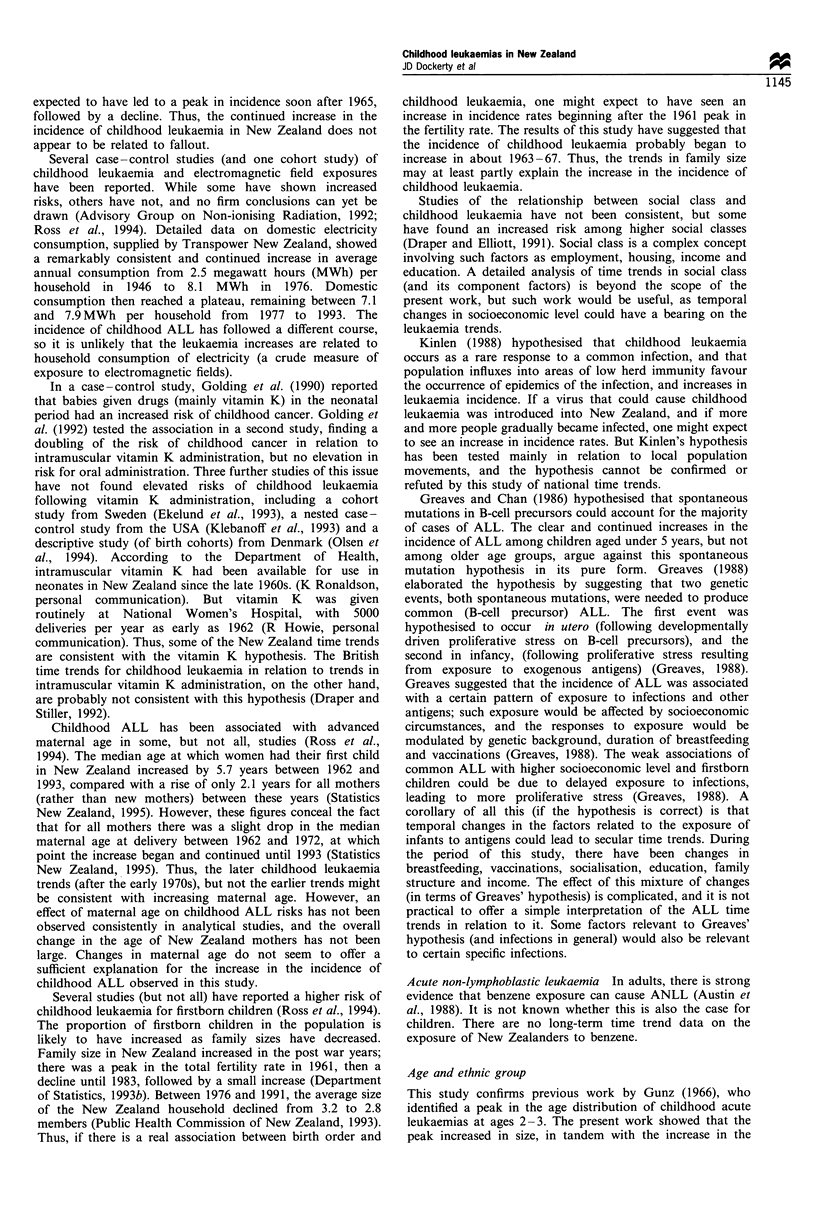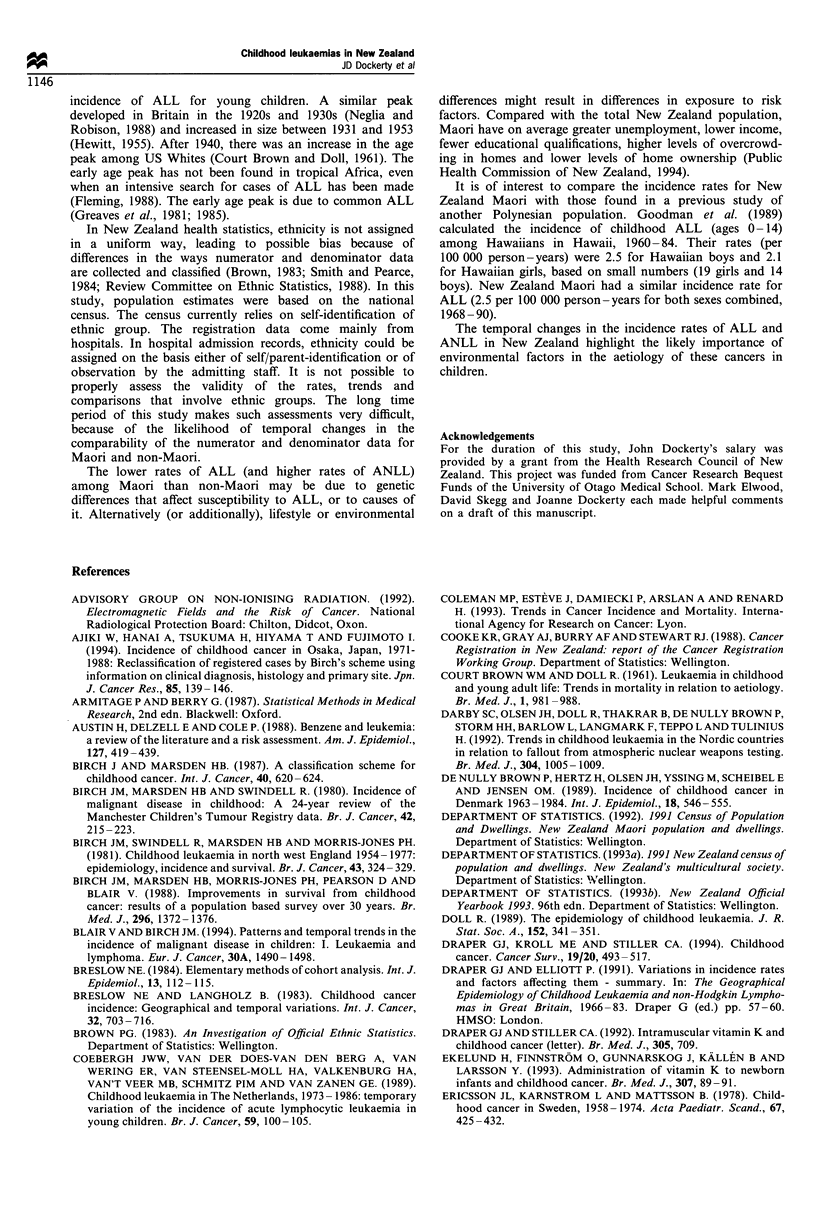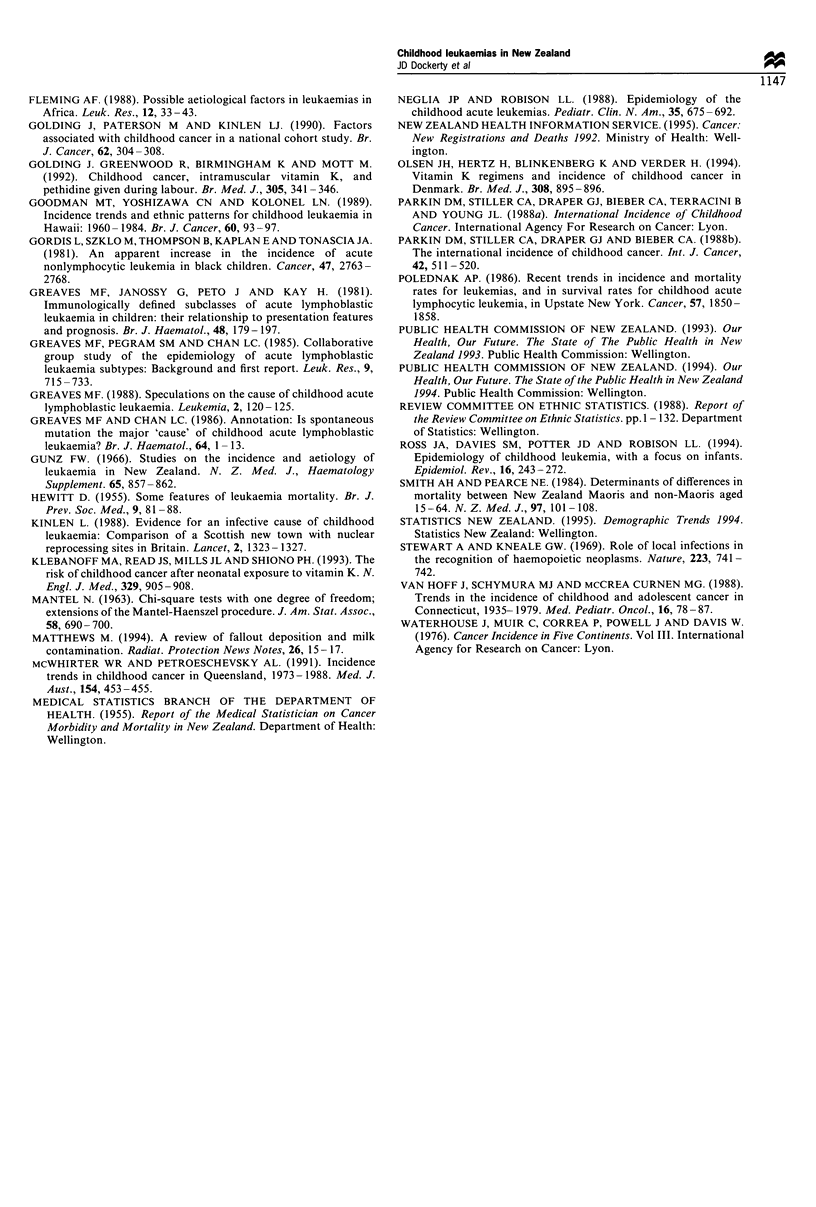# Childhood leukaemias in New Zealand: time trends and ethnic differences.

**DOI:** 10.1038/bjc.1996.219

**Published:** 1996-05

**Authors:** J. D. Dockerty, B. Cox, M. G. Cockburn

**Affiliations:** Department of Preventive and Social Medicine, University of Otago Medical School, Dunedin, New Zealand.

## Abstract

Registrations from the New Zealand Cancer Registry were used to examine time trends in the incidence of leukaemias among children aged 0-14. There was a statistically significant increase in the incidence of leukaemia among children aged 0-4 during 1953-57 to 1988-90. In this age group, the recorded incidence rate increased from 4.89 per 100,000 person-years in 1953-57 to 7.92 in 1988-90. During 1973-77 to 1988-90 (and probably in earlier years), the increase was due to an increase in acute lymphoblastic leukaemia (ALL). The trends were unlikely to be due to changes in diagnosis or case ascertainment. The childhood leukaemia trends might be related to trends in family size, maternal age, socioeconomic level or exposure to infections. However, there are uncertainties about the importance of these factors or about their trends. The incidence of acute non-lymphoblastic leukaemia (ANLL) decreased between 1968-72 and 1988-90. The time trends highlight the likely importance of environmental factors in the aetiology of childhood leukaemias in New Zealand. The risk of ALL was lower in the Maori than in the non-Maori population (relative risk Maori/non-Maori 0.74). The risk of ANLL was higher among Maori (relative risk 1.84).


British Journal of Cancer (1996) 73, 1141-1147
© 1996 Stockton Press All rights reserved 0007-0920/96 $12.00

### Childhood leukaemias in New Zealand: time trends and ethnic differences

JD Dockerty, B Cox and MG Cockburn

*Department of Preventive and Social Medicine, University of Otago Medical School, PO Box 913, Dunedin, New Zealand.*

**Summary** Registrations from the New Zealand Cancer Registry were used to examine time trends in the incidence of leukaemias among children aged 0-14. There was a statistically significant increase in the incidence of leukaemia among children aged 0-4 during 1953-57 to 1988-90. In this age group, the recorded incidence rate increased from 4.89 per 100 000 person-years in 1953-57 to 7.92 in 1988-90. During 1973-77 to 1988-90 (and probably in earlier years), the increase was due to an increase in acute lymphoblastic leukaemia (ALL). The trends were unlikely to be due to changes in diagnosis or case ascertainment. The childhood leukaemia trends might be related to trends in family size, maternal age, socioeconomic level or exposure to infections. However, there are uncertainties about the importance of these factors or about their trends. The incidence of acute non-lymphoblastic leukaemia (ANLL) decreased between 1968-72 and 1988-90. The time trends highlight the likely importance of environmental factors in the aetiology of childhood leukaemias in New Zealand. The risk of ALL was lower in the Maori than in the non-Maori population (relative risk Maori/non-Maori 0.74). The risk of ANLL was higher among Maori (relative risk 1.84).

**Keywords:** leukaemia; child; epidemiology; trend; aetiology; incidence

Information on the incidence of childhood leukaemias has been collected by the New Zealand Cancer Registry since 1948. Until 1972, the Registry was mainly public hospital based, but since then it has included registrations from private hospitals, death certificates and autopsy reports (Cooke *et al.*, 1988). The Registry was regarded as being truly population based from 1974 (Cooke *et al.*, 1988). Childhood leukaemias are serious diseases, and affected children would usually have been admitted to hospital. Data on completeness suggest that registration of lymphatic and haematopoietic cancers was nearly complete from about 1953-55 (Medical Statistics Branch of the Department of Health, 1955).

At the 1991 census, 14% of the childhood (ages 0-14) residents of New Zealand were reported as being of solely Maori ethnicity, and a further 7% were reported as being of mixed New Zealand Maori ethnicity (Department of Statistics, 1992). Children of sole Pacific Island ethnic groups made up 5% of the total resident childhood population (Department of Statistics, 1993*a*). Cancer registration data for Maori are thought to have been more closely related to sole Maori ethnicity than to mixed (plus sole) Maori ethnicity (New Zealand Health Information Service, 1995). 'Non-Maori' has been used in cancer registry publications to refer to the rest of the population, which is predominantly of British origin. Pacific Islanders form a small part of the non-Maori population.

In a review of 49 countries, the highest age-standardised incidence rate for childhood acute lymphoblastic leukaemia (ALL) (ages 0-14) was found in Costa Rica (4.5 per 100 000 person-years) (Parkin *et al.*, 1988*a*). The rates of childhood ALL in New Zealand were 3.2 per 100000 person-years among non-Maori and 1.3 per 100 000 person-years among Maori (Parkin *et al.*, 1988*a*). New Zealand Maori had the highest rate of childhood ANLL of any population studied (Parkin *et al.*, 1988*b*), while non-Maori New Zealanders had the highest rate of any predominantly white population (Parkin *et al.*, 1988*a*).

Three studies of time trends in multiple countries have included data from the New Zealand Cancer Registry, and have given different results. Breslow and Langholz (1983) found no significant trends in the incidence of childhood leukaemia for Maori or non-Maori over 1962-66 to 1972-76. Draper *et al.* (1994) calculated the cumulative incidence of combined childhood leukaemias among non-Maori as 66.0 (per 100 000) in 1972-76, with this decreasing to 62.1 in 1978-82, then increasing to 79.0 in 1983-87. Statistical tests for trends were not presented. Coleman *et al.* (1993) found that boys in New Zealand (but not girls) had a statistically significant increase in the cumulative risk of childhood leukaemia during the period 1965-85. The present study examines the recorded incidence of childhood leukaemia in New Zealand between 1948 and 1990.

### Materials and methods

Childhood (ages 0-14 years) leukaemia registrations were obtained from the New Zealand Cancer Registry for each year during 1948-90. For the division into the different types [ALL, acute non-lymphoblastic leukaemia (ANLL), other and unspecified leukaemias] only the period 1968-90 was used. The International Classification of Diseases (ICD) codes used for the leukaemia registrations in this study were decided after discussions with haematologists and Cancer Registry staff, and after referring to the literature. In classifying the leukaemias, the aim was to ensure comparability across the time periods studied. During 1948-67, the combined leukaemias were represented by the following ICD-7 codes: 204.0, 204.1, 204.2, 204.3 and 204.4. During 1968-77, the different types of childhood leukaemia were assigned to the following ICD-8 codes: ALL 204.0, 207.0 and 204.9; ANLL 205.0, 206.0 and 207.2; 'other and unspecified' 204.1, 205.1, 206.1, 206.9, 207.1 and 207.9. During 1978-87 (ICD-9) and 1988-90 (ICD-9 CM), the codes were: ALL 204.0, 204.8, 204.9 and 208.0; ANLL 205.0, 206.0, 207.0, 207.2, 205.3 and 206.8; 'other and unspecified' 204.1, 204.2, 205.1, 205.2, 205.8, 205.9, 206.1, 206.2, 206.9, 208.1, 208.2, 208.8 and 208.9. Childhood tumours such as acute unspecified leukaemias were classified under acute lymphoblastic leukaemia because of the high likelihood that most were in this category in earlier years. The study was restricted to children who had New Zealand residential addresses at registration.

The correct birth date was available for 380 of the 444 children diagnosed between 1976 and 1987 as their birth certificates were being used in another study. The ages of 27 children were changed on the basis of the more accurate birthdates. For eight children (all with ALL), the change was sufficient to alter which 5 year age group the child was in. For one child, the corrected age was over 14 years, so the

Correspondence: J Dockerty
Received 20 June 1995; revised 14 November 1995; accepted 23 November 1995

child was excluded. For five of the eight, the corrections shifted the age group from that of 5–9 to 0–4. For one, the shift was from ages 10–14 to ages 0–4, and for the remaining child, the shift was from ages 10–14 to 5–9.

The numbers of deaths from childhood leukaemia (1948–90) were obtained from the New Zealand Health Information Service (for 1948 and 1949); and from annual publications of the Ministry of Health.

Annual mean population estimates, based on national census data, were used for the calculation of age-specific and age-standardised rates. Registration rates were calculated for all the leukaemias combined (1948–90); and for ALL, ANLL, and other and unspecified leukaemias (1968–90). Five year age groups were used, and the standard was the world standard population (Waterhouse *et al.*, 1976). The rates were calculated using pooled quinquennial periods, except that the last period was truncated to 3 years (1988–90). Rates were calculated for both sexes, Maori and non-Maori, and the total population. For combined leukaemias and ALL, the age group 0–4 was further subdivided, into ages 'under 1 year' and '1–4 years'. Trends in the quinquennial age-specific and age-standardised rates, and heterogeneity among the age-specific rates, were examined (Mantel, 1963; Armitage and Berry, 1987). The 95% confidence intervals for the age-standardised rates were based on the binomial approximation (Armitage and Berry, 1987). Age-specific incidence rates were calculated for individual years of age using pooled data (1981–90 for ALL and 1968–90 for ANLL). Relative risks for sex and ethnic group were also calculated, using pooled data from 1968–90. These were age-adjusted relative risks with 95% confidence intervals, calculated using the formulae described by Breslow (1984).

### Results

The number of children registered with leukaemia of any type during 1948–90 was 1409, among whom ALL (as defined in Materials and methods) was the commonest type. Of the 851 registrations for leukaemias during 1968–90, 633 (74%) were for ALL, 179 (21%) for ANLL and 39 (5%) for other and unspecified leukaemias. Ninety-five per cent of those classified as ALL had ICD codes corresponding to acute lymphatic or acute lymphoid leukaemias. Five per cent had ICD codes corresponding to other or unspecified lymphatic or acute leukaemias. In the first 5 years (1948–52), the ratio of registrations to deaths was 0.86 (Table I). In every other period, registrations outnumbered deaths, and the ratio increased continually, from 1.03 in 1953–57 to 2.62 in 1988–90.

### *Trends in the incidence of combined leukaemias*

In view of the incomplete registrations during 1948–52 (Table I), the presented results and the tests for trend for combined leukaemias were restricted to the period 1953–57 to 1988–90. There was a continued increase in the recorded incidence of combined leukaemias during 1953–57 to 1968–72, then a drop from 1968–72 to 1973–77, followed by a further increase from 1973–77 to 1983–87 (to a higher rate than before), with similar rates in 1983–87 and 1988–90 (Figure 1 and Table II).

In the total population, for all leukaemias combined, there was a significant increase in the age-standardised rates during 1953–57 to 1988–90 ($P=0.02$). The annual percentage increase in the rate, relative to the average rate, was 0.81% per year. There was significant heterogeneity among the rates in the different age groups ($P=0.02$). Children aged under 5 years had a highly significant increase ($P=0.0004$), which explained the trend in the age-standardised rates (Table II), while no overall trends were found for children aged 5–9 and 10–14. The increase in incidence for children under 5 years of age was present among boys ($P=0.01$), girls ($P=0.01$), and non-Maori ($P=0.0003$). For age groups 5–9 and 10–14, there were no overall trends in boys or girls or among non-Maori. For Maori, there was no overall statistical trend in the rates among those aged under 5 years, and for ages 5–9 and 10–14 there were too few Maori children with leukaemia to allow interpretation of the trends.

### *Trends in the incidence of ALL and ANLL*

Different trends in incidence were observed for ALL and ANLL (Figure 1 and Table III). The age-standardised incidence rate of ALL decreased from 1968–72 to 1973–77, then increased steadily in each quinquennium until 1988–90 (the rates were 3.22, 2.74, 3.22, 3.67 and 4.10 per 100 000 person-years). The incidence of ANLL decreased steadily between 1968–72 and 1978–82, then increased in 1983–87, and decreased to a low point in 1988–90 (the rates were 1.13, 0.97, 0.78, 0.92 and 0.59 per 100 000 person-years).

For ALL, the trend tests showed that the increase in the age-standardised rate was significant (1968–72 to 1988–90, $P=0.02$) and there was no significant departure from

**Figure 1** Age-standardised registration rates for childhood leukaemias in New Zealand, ages 0–14. ■, All leukaemias combined; □, acute lymphoblastic leukaemia; ●, acute non-lymphoblastic leukaemia; ○, other and unspecified leukaemias.

**Table I** Childhood leukaemia registrations and deaths[a] in New Zealand, 1948-90: all leukaemias combined, for ages 0–14

| *Time period* | *Number of registrations* | *Number of deaths* | *Ratio of registrations to deaths* |
|---|---|---|---|
| 1948–52 | 90 | 105 | 0.86 |
| 1953–57 | 126 | 122 | 1.03 |
| 1958–62 | 151 | 144 | 1.05 |
| 1963–67 | 191 | 160 | 1.19 |
| 1968–72 | 207 | 159 | 1.30 |
| 1973–77 | 175 | 134 | 1.31 |
| 1978–82 | 169 | 91 | 1.86 |
| 1983–87 | 190 | 104 | 1.83 |
| 1988–90 | 110 | 42 | 2.62 |

[a]In 1948, only non-Maori were included in the deaths. In all other years, Maori and non-Maori deaths were included.

**Table II** Childhood leukaemia registrations (numbers, and rates per 100 000 person-years) in New Zealand, 1953–90: combined leukaemias

| | Age group (years) | | | | | | | | |
|---|---|---|---|---|---|---|---|---|---|
| | 0–4 | | 5–9 | | 10–14 | | 0–14 | | |
| *Time period* | *No.* | *Rate* | *No.* | *Rate* | *No.* | *Rate* | *No.* | *Crude rate* | *ASR (with 95% CI)* |
| 1953–57 | 62 | 4.89 | 39 | 3.39 | 25 | 2.72 | 126 | 3.78 | 3.70 (3.41–3.99) |
| 1958–62 | 71 | 4.93 | 53 | 4.13 | 27 | 2.30 | 151 | 3.88 | 3.84 (3.57–4.11) |
| 1963–67 | 102 | 6.63 | 53 | 3.62 | 36 | 2.75 | 191 | 4.43 | 4.38 (4.11–4.66) |
| 1968–72 | 103 | 6.91 | 57 | 3.69 | 47 | 3.19 | 207 | 4.59 | 4.64 (4.36–4.93) |
| 1973–77 | 83 | 5.58 | 48 | 3.13 | 44 | 2.78 | 175 | 3.80 | 3.87 (3.61–4.12) |
| 1978–82 | 85 | 6.62 | 46 | 3.12 | 38 | 2.50 | 169 | 3.95 | 4.13 (3.85–4.41) |
| 1983–87 | 100 | 7.92 | 43 | 3.33 | 47 | 3.18 | 190 | 4.71 | 4.87 (4.56–5.18) |
| 1988–90 | 63 | 7.92 | 29 | 3.87 | 18 | 2.30 | 110 | 4.73 | 4.78 (4.38–5.18) |

ASR, age-standardised rate; CI, confidence interval.

**Table III** Childhood leukaemia registrations (numbers and rates per 100 000 person-years) by age, sex, and ethnic group, 1968–90: by type of leukaemia

| | Age group (years) | | | | | | | Sex | | | | Ethnic group | | | |
|---|---|---|---|---|---|---|---|---|---|---|---|---|---|---|---|
| *Leukaemia type,* | 0–4 | | 5–9 | | 10–14 | | 0–14 | Boys | | Girls | | Maori | | Non-Maori | |
| *time period* | *No.* | *Rate* | *No.* | *Rate* | *No.* | *Rate* | *ASR* | *No.* | *ASR* | *No.* | *ASR* | *No.* | *ASR* | *No.* | *ASR* |
| ALL | | | | | | | | | | | | | | | |
| 1968–72 | 72 | 4.83 | 46 | 2.98 | 25 | 1.69 | 3.22 | 79 | 3.49 | 64 | 2.94 | 21 | 3.80 | 122 | 3.14 |
| 1973–77 | 60 | 4.04 | 34 | 2.22 | 30 | 1.89 | 2.74 | 79 | 3.45 | 45 | 2.01 | 5 | 0.88 | 119 | 3.02 |
| 1978–82 | 71 | 5.53 | 36 | 2.44 | 23 | 1.51 | 3.22 | 78 | 3.76 | 52 | 2.66 | 13 | 2.41 | 117 | 3.34 |
| 1983–87 | 81 | 6.42 | 31 | 2.40 | 30 | 2.03 | 3.67 | 90 | 4.58 | 52 | 2.71 | 13 | 2.63 | 129 | 3.83 |
| 1988–90 | 56 | 7.04 | 26 | 3.47 | 12 | 1.53 | 4.10 | 45 | 3.83 | 49 | 4.39 | 10 | 3.45 | 84 | 4.20 |
| ANLL | | | | | | | | | | | | | | | |
| 1968–72 | 23 | 1.54 | 9 | 0.58 | 19 | 1.29 | 1.13 | 30 | 1.30 | 21 | 0.96 | 7 | 1.28 | 44 | 1.11 |
| 1973–77 | 19 | 1.28 | 12 | 0.78 | 13 | 0.82 | 0.97 | 24 | 1.03 | 20 | 0.90 | 8 | 1.40 | 36 | 0.90 |
| 1978–82 | 12 | 0.93 | 9 | 0.61 | 12 | 0.79 | 0.78 | 21 | 0.97 | 12 | 0.58 | 11 | 1.94 | 22 | 0.60 |
| 1983–87 | 15 | 1.19 | 8 | 0.62 | 14 | 0.95 | 0.92 | 18 | 0.87 | 19 | 0.97 | 10 | 1.91 | 27 | 0.77 |
| 1988–90 | 5 | 0.63 | 3 | 0.40 | 6 | 0.77 | 0.59 | 9 | 0.74 | 5 | 0.44 | 2 | 0.70 | 12 | 0.57 |
| Other/unspecified | | | | | | | | | | | | | | | |
| 1968–72 | 8 | 0.54 | 2 | 0.13 | 3 | 0.20 | 0.29 | 9 | 0.40 | 4 | 0.18 | 2 | 0.36 | 11 | 0.28 |
| 1973–77 | 4 | 0.27 | 2 | 0.13 | 1 | 0.06 | 0.16 | 3 | 0.14 | 4 | 0.18 | 1 | 0.18 | 6 | 0.15 |
| 1978–82 | 2 | 0.16 | 1 | 0.07 | 3 | 0.20 | 0.14 | 4 | 0.19 | 2 | 0.08 | 2 | 0.40 | 4 | 0.10 |
| 1983–87 | 4 | 0.32 | 4 | 0.31 | 3 | 0.20 | 0.28 | 3 | 0.14 | 8 | 0.43 | 2 | 0.37 | 9 | 0.27 |
| 1988–90 | 2 | 0.25 | 0 | 0.00 | 0 | 0.00 | 0.09 | 1 | 0.08 | 1 | 0.09 | 1 | 0.35 | 1 | 0.05 |

ASR, age-standardised rate; ALL, acute lymphoblastic leukaemia; ANLL, acute non-lymphoblastic leukaemia.

linearity, although the rate during 1973–77 was lower than the rates in adjacent time periods. The annual percentage increase in the rate of ALL (1968–90), relative to the average rate, was 2.2% per year. The increase in total leukaemias among children aged under 5 years (Table II), at least during 1968–90, was due to a significant ($P=0.002$) increase in ALL for this age group (Table III). There were no overall trends in ALL incidence for ages 5–9 or 10–14.

During 1968–72 to 1988–90, boys aged under 5 years had a significant increase in incidence ($P=0.04$), and there was no significant departure from linearity. Girls aged under 5 years also had an increase in ALL incidence, but the trend was significantly non-linear (the incidence rate was 4.7 per 100 000 person-years in 1968–72, 2.6 in 1973–77, 4.9 in 1978–82, 4.2 in 1983–87 and 8.0 in 1988–90). Non-Maori aged under 5 years had a highly significant increase in the incidence of ALL ($P=0.004$).

In the total population, there were too few children aged under 1 year for the interpretation of time trends, though ALL incidence rates for ages under 1 year increased steadily in each quinquennium from 1968–72 to 1988–90. Children aged 1–4 experienced a significant increase in the incidence of ALL over the period ($P=0.007$).

For ANLL, there was a significant linear decrease in the incidence rates (1968–72 to 1988–90; Figure 1 and Table III). There was no significant heterogeneity among the different age groups. The annual percentage decrease, relative to the average rate, was 3.8% per year. In boys, no overall trend in the age-standardised rates was found, and in girls the trend tests could not be interpreted owing to small numbers. Among non-Maori, there was a significant linear decrease in the incidence of ANLL ($P=0.02$).

### *Age, sex and ethnic group*

For ALL, the age distribution by individual years of age showed a marked peak at ages 2–3 (the peak incidence rate was 9.3 per 100 000 person-years). Sequential data for combined leukaemias revealed that the size of the age peak increased during 1953–90. The early ALL age peak was marked for both Maori and non-Maori. The pooled (1968–90) ALL registration rates for each 5 year age group (per 100 000 person-years) were: ages 0–4 Maori 6.0, non-Maori 6.6; ages 5–9 Maori 1.6, non-Maori 2.9; and ages 10–14 Maori 1.0, non-Maori 1.8. For ANLL, although the total numbers were small, there were clearly high registration rates at ages 0–2, with lower rates at ages 3–14 years.

Compared with girls, boys had a greater risk of both types of leukaemia (statistically significant for ALL but not for ANLL, Table IV). Maori had a lower risk of ALL and a higher risk of ANLL than non-Maori (Table IV). The crude relative risks (boys *vs* girls and Maori *vs* non-Maori) were virtually identical to the age-adjusted relative risks.

### Discussion

The most interesting finding was the clear increase in leukaemia incidence rates for children aged 0–4 during 1953–57 to 1988–90 (Table II). During 1973–77 to 1988–90 (and probably in earlier years), this was due to an increase in ALL (Table III). The incidence of ANLL decreased during 1968–72 to 1988–90 (Figure 1, Table III). What are the explanations for these trends? Underdiagnosis due to masking by other causes of death such as pneumonia in the

Table IV Relative risks for sex and ethnicity, 1968–90: by type of leukaemia, for ages 0–14

| Leukaemia type | Age-standardised incidence rate per 100 000 person–years Boys | Girls | Age-adjusted relative risk boys/girls (95% CI) | Age-standardised incidence rate per 100 000 person–years Maori | Non-Maori | Age-adjusted relative risk Maori/non-Maori (95% CI) |
|---|---|---|---|---|---|---|
| Acute lymphoblastic | 3.95 | 2.90 | 1.36 (1.16–1.59) | 2.65 | 3.56 | 0.73 (0.57–0.95) |
| Acute non-lymphoblastic | 1.01 | 0.84 | 1.27 (0.94–1.70) | 1.55 | 0.83 | 1.84 (1.29–2.62) |

preantibiotic age (Stewart and Kneale, 1969) may have had an effect on the accuracy of diagnosis in the early years, but could not account for the continuation of the trends beyond the introduction of antibiotics.

The childhood leukaemias in this study were classified into three types (ALL, ANLL and 'other and unspecified'), based on ICD site codes. Childhood cancers are better suited to classifications based on morphology rather than site. The Birch and Marsden (1987) classification separates the childhood leukaemias into five types (acute lymphocytic, other lymphoid, acute non-lymphocytic, chronic myeloid and 'other and unspecified'), based on ICD-O (oncology) morphology codes. ICD-O codes have only been used by the New Zealand Cancer Registry since 1978. Site-based classifications were used in this study, and the aim was to enable the two main types of childhood leukaemia (ALL and ANLL) to be distinguished, from 1968 onwards.

Histological classification of the separate types of leukaemia is considered to have been relatively accurate since the late 1960s in New Zealand (personal communications from D Becroft, C Beresford and J Carter). There may have been some under-reporting of the myeloid leukaemias, because specialists might have expected children to have lymphoid leukaemias and have classified them as such. Misclassification is likely to have had a moderating effect on the observed trends, rather than an effect that would lead to spurious trends. If anything, one would have expected a decrease in ALL and an increase in ANLL rather than the converse, which has been found. The trends are therefore unlikely to be related to changes in the diagnosis of the two main types of childhood leukaemia.

Comparable analyses have shown that there were no significant time trends in the registration rates of the childhood non-Hodgkin's lymphomas in New Zealand during 1953–90 (work in progress). Thus, the leukaemia trends do not seem to be due to diagnostic shifts between ALL and the non-Hodgkin's lymphomas.

One indication of the quality of a cancer registry is the proportion of cases for which the diagnosis has a histological basis, assuming that there is good completeness of registration, and that the histology is accurate. During 1970–79, 100% of the children with ALL and ANLL on the New Zealand Cancer Register had a histological basis for diagnosis (Parkin et al., 1988a). There is no published information on this for childhood leukaemias in earlier periods.

The 5 year survival from childhood leukaemia in Britain was very low (2%) for children diagnosed during 1954–63 (Birch et al., 1988), and is not likely to have been higher in New Zealand at the time. The recording of more registrations than deaths in 1953–57 and in 1958–62 is evidence that case ascertainment was nearly complete, even during those periods. Thus, improvements in ascertainment do not explain the trends. The subsequent increases in the ratio of registrations to deaths (Table I) reflect improvements in treatment over time.

Researchers from other countries have reported increases in the incidence of ALL among children aged 0–4 or 1–4. These include studies in Britain 1953–91 (Draper et al., 1994); north-west England 1954–77 (Birch et al., 1980; Birch et al., 1981); the Netherlands 1973–86 (temporary increase 1974–82) (Coebergh et al., 1989); and Connecticut 1935–79 (van Hoff et al., 1988). The data from north-west England were recently updated to include 1954–88, and although there was an overall increase for ages 0–14, there were no significant time trends for boys or girls aged 1–4 (Blair and Birch, 1994). A study of childhood ALL in Baltimore 1960–74 (Gordis et al., 1981) did not show an increase for children aged 1–4. Two other studies (limited to all leukaemias combined) have shown increases among young children, including a study from Sweden 1958–74 (Ericsson et al., 1978), and one from Denmark 1943–84 (de Nully Brown et al., 1989). A study of combined leukaemias in upstate New York (1969–80) did not record a significant change in incidence among boys or girls aged under 5 years (Polednak, 1986). The New Zealand increase was one of the more obvious secular trends among young children in latter decades.

For childhood ANLL, temporal decreases in incidence rates have been reported in two other studies: one in Britain 1962–91 (Draper et al., 1994), and another in Japan (Osaka) 1971–88 (Ajiki et al., 1994). The decrease in Osaka may have been partly due to changes in the diagnosis or classification of ALL and ANLL, although the authors were of the opinion that most of the leukaemia changes they observed were real (Ajiki et al., 1994). Significant increases have been reported in Queensland 1973–88 (McWhirter and Petroeschevsky, 1991); and among black children in Baltimore 1965–74 (Gordis et al., 1981). Incidence rates of childhood ANLL in the Netherlands 1973–86 are reported to have been relatively constant (Coebergh et al., 1989). In north-west England 1954–88, there was no significant trend in the incidence of childhood ANLL (Blair and Birch, 1994).

*Relevance of time trends to aetiological factors and hypotheses*

*ALL* Because ALL was by far the commonest type, the trends in combined leukaemias should mostly reflect trends in the incidence of ALL. Up until 1953 (at least), childhood leukaemia was 'rapidly and universally fatal', and mortality and incidence were equivalent (Draper et al., 1994). If the correct number of incident cases of childhood leukaemia for 1948–52 was 105 (deaths) and not 90 (registrations), the crude incidence rate for 1948–52 would be 3.77 per 100 000 person–years, little different from 3.78 in 1953–57 (Table II). The increase for young children in 1958–62 was minor, but there were clear increases in 1963–67 and 1968–72 (Table II). Incidence dropped between 1968–72 and 1973–77, then increased again in 1978–82 and 1983–87. Thus the combined leukaemia results suggest that the incidence of ALL in young children began to increase clearly in 1963–67.

The risks of childhood leukaemia from ionising radiation exposure have been established (Doll, 1989). In New Zealand, we have no nuclear power stations or reprocessing plants. A study of childhood leukaemia incidence rates in Nordic countries in relation to fallout from nuclear weapons testing found no strong overall childhood leukaemia trends (Darby et al., 1992). However, the rates of childhood leukaemia were slightly higher in the late 1960s, when the effect would have been greatest (Darby et al., 1992). Fallout from nuclear weapons testing has been monitored in New Zealand by the National Radiation Laboratory, and 'concentrations of strontium-90 and caesium-137 in cows' milk peaked in 1965 ..... and had decreased to the limits of detection by 1986', (Matthews, 1994). Initially (after the 1965 peak), the average half-life of each radionuclide was less than 2 years (Matthews, 1994). If fallout had a material effect on leukaemia in young children in New Zealand, it would be expected to have led to a peak in incidence soon after 1965, followed by a decline. Thus, the continued increase in the incidence of childhood leukaemia in New Zealand does not appear to be related to fallout.

Several case–control studies (and one cohort study) of childhood leukaemia and electromagnetic field exposures have been reported. While some have shown increased risks, others have not, and no firm conclusions can yet be drawn (Advisory Group on Non-ionising Radiation, 1992; Ross *et al.*, 1994). Detailed data on domestic electricity consumption, supplied by Transpower New Zealand, showed a remarkably consistent and continued increase in average annual consumption from 2.5 megawatt hours (MWh) per household in 1946 to 8.1 MWh in 1976. Domestic consumption then reached a plateau, remaining between 7.1 and 7.9 MWh per household from 1977 to 1993. The incidence of childhood ALL has followed a different course, so it is unlikely that the leukaemia increases are related to household consumption of electricity (a crude measure of exposure to electromagnetic fields).

In a case–control study, Golding *et al.* (1990) reported that babies given drugs (mainly vitamin K) in the neonatal period had an increased risk of childhood cancer. Golding *et al.* (1992) tested the association in a second study, finding a doubling of the risk of childhood cancer in relation to intramuscular vitamin K administration, but no elevation in risk for oral administration. Three further studies of this issue have not found elevated risks of childhood leukaemia following vitamin K administration, including a cohort study from Sweden (Ekelund *et al.*, 1993), a nested case–control study from the USA (Klebanoff *et al.*, 1993) and a descriptive study (of birth cohorts) from Denmark (Olsen *et al.*, 1994). According to the Department of Health, intramuscular vitamin K had been available for use in neonates in New Zealand since the late 1960s. (K Ronaldson, personal communication). But vitamin K was given routinely at National Women's Hospital, with 5000 deliveries per year as early as 1962 (R Howie, personal communication). Thus, some of the New Zealand time trends are consistent with the vitamin K hypothesis. The British time trends for childhood leukaemia in relation to trends in intramuscular vitamin K administration, on the other hand, are probably not consistent with this hypothesis (Draper and Stiller, 1992).

Childhood ALL has been associated with advanced maternal age in some, but not all, studies (Ross *et al.*, 1994). The median age at which women had their first child in New Zealand increased by 5.7 years between 1962 and 1993, compared with a rise of only 2.1 years for all mothers (rather than new mothers) between these years (Statistics New Zealand, 1995). However, these figures conceal the fact that for all mothers there was a slight drop in the median maternal age at delivery between 1962 and 1972, at which point the increase began and continued until 1993 (Statistics New Zealand, 1995). Thus, the later childhood leukaemia trends (after the early 1970s), but not the earlier trends might be consistent with increasing maternal age. However, an effect of maternal age on childhood ALL risks has not been observed consistently in analytical studies, and the overall change in the age of New Zealand mothers has not been large. Changes in maternal age do not seem to offer a sufficient explanation for the increase in the incidence of childhood ALL observed in this study.

Several studies (but not all) have reported a higher risk of childhood leukaemia for firstborn children (Ross *et al.*, 1994). The proportion of firstborn children in the population is likely to have increased as family sizes have decreased. Family size in New Zealand increased in the post war years; there was a peak in the total fertility rate in 1961, then a decline until 1983, followed by a small increase (Department of Statistics, 1993*b*). Between 1976 and 1991, the average size of the New Zealand household declined from 3.2 to 2.8 members (Public Health Commission of New Zealand, 1993). Thus, if there is a real association between birth order and childhood leukaemia, one might expect to have seen an increase in incidence rates beginning after the 1961 peak in the fertility rate. The results of this study have suggested that the incidence of childhood leukaemia probably began to increase in about 1963–67. Thus, the trends in family size may at least partly explain the increase in the incidence of childhood leukaemia.

Studies of the relationship between social class and childhood leukaemia have not been consistent, but some have found an increased risk among higher social classes (Draper and Elliott, 1991). Social class is a complex concept involving such factors as employment, housing, income and education. A detailed analysis of time trends in social class (and its component factors) is beyond the scope of the present work, but such work would be useful, as temporal changes in socioeconomic level could have a bearing on the leukaemia trends.

Kinlen (1988) hypothesised that childhood leukaemia occurs as a rare response to a common infection, and that population influxes into areas of low herd immunity favour the occurrence of epidemics of the infection, and increases in leukaemia incidence. If a virus that could cause childhood leukaemia was introduced into New Zealand, and if more and more people gradually became infected, one might expect to see an increase in incidence rates. But Kinlen's hypothesis has been tested mainly in relation to local population movements, and the hypothesis cannot be confirmed or refuted by this study of national time trends.

Greaves and Chan (1986) hypothesised that spontaneous mutations in B-cell precursors could account for the majority of cases of ALL. The clear and continued increases in the incidence of ALL among children aged under 5 years, but not among older age groups, argue against this spontaneous mutation hypothesis in its pure form. Greaves (1988) elaborated the hypothesis by suggesting that two genetic events, both spontaneous mutations, were needed to produce common (B-cell precursor) ALL. The first event was hypothesised to occur *in utero* (following developmentally driven proliferative stress on B-cell precursors), and the second in infancy, (following proliferative stress resulting from exposure to exogenous antigens) (Greaves, 1988). Greaves suggested that the incidence of ALL was associated with a certain pattern of exposure to infections and other antigens; such exposure would be affected by socioeconomic circumstances, and the responses to exposure would be modulated by genetic background, duration of breastfeeding and vaccinations (Greaves, 1988). The weak associations of common ALL with higher socioeconomic level and firstborn children could be due to delayed exposure to infections, leading to more proliferative stress (Greaves, 1988). A corollary of all this (if the hypothesis is correct) is that temporal changes in the factors related to the exposure of infants to antigens could lead to secular time trends. During the period of this study, there have been changes in breastfeeding, vaccinations, socialisation, education, family structure and income. The effect of this mixture of changes (in terms of Greaves' hypothesis) is complicated, and it is not practical to offer a simple interpretation of the ALL time trends in relation to it. Some factors relevant to Greaves' hypothesis (and infections in general) would also be relevant to certain specific infections.

*Acute non-lymphoblastic leukaemia* In adults, there is strong evidence that benzene exposure can cause ANLL (Austin *et al.*, 1988). It is not known whether this is also the case for children. There are no long-term time trend data on the exposure of New Zealanders to benzene.

### *Age and ethnic group*

This study confirms previous work by Gunz (1966), who identified a peak in the age distribution of childhood acute leukaemias at ages 2–3. The present work showed that the peak increased in size, in tandem with the increase in the incidence of ALL for young children. A similar peak developed in Britain in the 1920s and 1930s (Neglia and Robison, 1988) and increased in size between 1931 and 1953 (Hewitt, 1955). After 1940, there was an increase in the age peak among US Whites (Court Brown and Doll, 1961). The early age peak has not been found in tropical Africa, even when an intensive search for cases of ALL has been made (Fleming, 1988). The early age peak is due to common ALL (Greaves *et al.*, 1981; 1985).

In New Zealand health statistics, ethnicity is not assigned in a uniform way, leading to possible bias because of differences in the ways numerator and denominator data are collected and classified (Brown, 1983; Smith and Pearce, 1984; Review Committee on Ethnic Statistics, 1988). In this study, population estimates were based on the national census. The census currently relies on self-identification of ethnic group. The registration data come mainly from hospitals. In hospital admission records, ethnicity could be assigned on the basis either of self/parent-identification or of observation by the admitting staff. It is not possible to properly assess the validity of the rates, trends and comparisons that involve ethnic groups. The long time period of this study makes such assessments very difficult, because of the likelihood of temporal changes in the comparability of the numerator and denominator data for Maori and non-Maori.

The lower rates of ALL (and higher rates of ANLL) among Maori than non-Maori may be due to genetic differences that affect susceptibility to ALL, or to causes of it. Alternatively (or additionally), lifestyle or environmental differences might result in differences in exposure to risk factors. Compared with the total New Zealand population, Maori have on average greater unemployment, lower income, fewer educational qualifications, higher levels of overcrowding in homes and lower levels of home ownership (Public Health Commission of New Zealand, 1994).

It is of interest to compare the incidence rates for New Zealand Maori with those found in a previous study of another Polynesian population. Goodman *et al.* (1989) calculated the incidence of childhood ALL (ages 0–14) among Hawaiians in Hawaii, 1960–84. Their rates (per 100 000 person-years) were 2.5 for Hawaiian boys and 2.1 for Hawaiian girls, based on small numbers (19 girls and 14 boys). New Zealand Maori had a similar incidence rate for ALL (2.5 per 100 000 person-years for both sexes combined, 1968–90).

The temporal changes in the incidence rates of ALL and ANLL in New Zealand highlight the likely importance of environmental factors in the aetiology of these cancers in children.

**Acknowledgements**
For the duration of this study, John Dockerty's salary was provided by a grant from the Health Research Council of New Zealand. This project was funded from Cancer Research Bequest Funds of the University of Otago Medical School. Mark Elwood, David Skegg and Joanne Dockerty each made helpful comments on a draft of this manuscript.

### References

ADVISORY GROUP ON NON-IONISING RADIATION. (1992). *Electromagnetic Fields and the Risk of Cancer*. National Radiological Protection Board: Chilton, Didcot, Oxon.

AJIKI W, HANAI A, TSUKUMA H, HIYAMA T AND FUJIMOTO I. (1994). Incidence of childhood cancer in Osaka, Japan, 1971-1988: Reclassification of registered cases by Birch's scheme using information on clinical diagnosis, histology and primary site. *Jpn. J. Cancer Res.*, **85**, 139–146.

ARMITAGE P AND BERRY G. (1987). *Statistical Methods in Medical Research*, 2nd edn. Blackwell: Oxford.

AUSTIN H, DELZELL E AND COLE P. (1988). Benzene and leukemia: a review of the literature and a risk assessment. *Am. J. Epidemiol.*, **127**, 419–439.

BIRCH J AND MARSDEN HB. (1987). A classification scheme for childhood cancer. *Int. J. Cancer*, **40**, 620–624.

BIRCH JM, MARSDEN HB AND SWINDELL R. (1980). Incidence of malignant disease in childhood: A 24-year review of the Manchester Children's Tumour Registry data. *Br. J. Cancer*, **42**, 215–223.

BIRCH JM, SWINDELL R, MARSDEN HB AND MORRIS-JONES PH. (1981). Childhood leukaemia in north west England 1954–1977: epidemiology, incidence and survival. *Br. J. Cancer*, **43**, 324–329.

BIRCH JM, MARSDEN HB, MORRIS-JONES PH, PEARSON D AND BLAIR V. (1988). Improvements in survival from childhood cancer: results of a population based survey over 30 years. *Br. Med. J.*, **296**, 1372–1376.

BLAIR V AND BIRCH JM. (1994). Patterns and temporal trends in the incidence of malignant disease in children: I. Leukaemia and lymphoma. *Eur. J. Cancer*, **30A**, 1490–1498.

BRESLOW NE. (1984). Elementary methods of cohort analysis. *Int. J. Epidemiol.*, **13**, 112–115.

BRESLOW NE AND LANGHOLZ B. (1983). Childhood cancer incidence: Geographical and temporal variations. *Int. J. Cancer*, **32**, 703–716.

BROWN PG. (1983). *An Investigation of Official Ethnic Statistics*. Department of Statistics: Wellington.

COEBERGH JWW, VAN DER DOES-VAN DEN BERG A, VAN WERING ER, VAN STEENSEL-MOLL HA, VALKENBURG HA, VAN'T VEER MB, SCHMITZ PIM AND VAN ZANEN GE. (1989). Childhood leukaemia in The Netherlands, 1973–1986: temporary variation of the incidence of acute lymphocytic leukaemia in young children. *Br. J. Cancer*, **59**, 100–105.

COLEMAN MP, ESTÈVE J, DAMIECKI P, ARSLAN A AND RENARD H. (1993). Trends in Cancer Incidence and Mortality. International Agency for Research on Cancer: Lyon.

COOKE KR, GRAY AJ, BURRY AF AND STEWART RJ. (1988). *Cancer Registration in New Zealand: report of the Cancer Registration Working Group*. Department of Statistics: Wellington.

COURT BROWN WM AND DOLL R. (1961). Leukaemia in childhood and young adult life: Trends in mortality in relation to aetiology. *Br. Med. J.*, **1**, 981–988.

DARBY SC, OLSEN JH, DOLL R, THAKRAR B, DE NULLY BROWN P, STORM HH, BARLOW L, LANGMARK F, TEPPO L AND TULINIUS H. (1992). Trends in childhood leukaemia in the Nordic countries in relation to fallout from atmospheric nuclear weapons testing. *Br. Med. J.*, **304**, 1005–1009.

DE NULLY BROWN P, HERTZ H, OLSEN JH, YSSING M, SCHEIBEL E AND JENSEN OM. (1989). Incidence of childhood cancer in Denmark 1963–1984. *Int. J. Epidemiol.*, **18**, 546–555.

DEPARTMENT OF STATISTICS. (1992). *1991 Census of Population and Dwellings. New Zealand Maori population and dwellings*. Department of Statistics: Wellington.

DEPARTMENT OF STATISTICS. (1993*a*). *1991 New Zealand census of population and dwellings. New Zealand's multicultural society*. Department of Statistics: Wellington.

DEPARTMENT OF STATISTICS. (1993*b*). *New Zealand Official Yearbook 1993*. 96th edn. Department of Statistics: Wellington.

DOLL R. (1989). The epidemiology of childhood leukaemia. *J. R. Stat. Soc. A.*, **152**, 341–351.

DRAPER GJ, KROLL ME AND STILLER CA. (1994). Childhood cancer. *Cancer Surv.*, **19/20**, 493–517.

DRAPER GJ AND ELLIOTT P. (1991). Variations in incidence rates and factors affecting them - summary. In: *The Geographical Epidemiology of Childhood Leukaemia and non-Hodgkin Lymphomas in Great Britain*, 1966–83. Draper G (ed.) pp. 57–60. HMSO: London.

DRAPER GJ AND STILLER CA. (1992). Intramuscular vitamin K and childhood cancer (letter). *Br. Med. J.*, **305**, 709.

EKELUND H, FINNSTRÖM O, GUNNARSKOG J, KÄLLÉN B AND LARSSON Y. (1993). Administration of vitamin K to newborn infants and childhood cancer. *Br. Med. J.*, **307**, 89–91.

ERICSSON JL, KARNSTROM L AND MATTSSON B. (1978). Childhood cancer in Sweden, 1958–1974. *Acta Paediatr. Scand.*, **67**, 425–432.

FLEMING AF. (1988). Possible aetiological factors in leukaemias in Africa. *Leuk. Res.*, **12**, 33-43.
GOLDING J, PATERSON M AND KINLEN LJ. (1990). Factors associated with childhood cancer in a national cohort study. *Br. J. Cancer*, **62**, 304-308.
GOLDING J. GREENWOOD R, BIRMINGHAM K AND MOTT M. (1992). Childhood cancer, intramuscular vitamin K, and pethidine given during labour. *Br. Med. J.*, **305**, 341-346.
GOODMAN MT, YOSHIZAWA CN AND KOLONEL LN. (1989). Incidence trends and ethnic patterns for childhood leukaemia in Hawaii: 1960-1984. *Br. J. Cancer*, **60**, 93-97.
GORDIS L, SZKLO M, THOMPSON B, KAPLAN E AND TONASCIA JA. (1981). An apparent increase in the incidence of acute nonlymphocytic leukemia in black children. *Cancer*, **47**, 2763-2768.
GREAVES MF, JANOSSY G, PETO J AND KAY H. (1981). Immunologically defined subclasses of acute lymphoblastic leukaemia in children: their relationship to presentation features and prognosis. *Br. J. Haematol.*, **48**, 179-197.
GREAVES MF, PEGRAM SM AND CHAN LC. (1985). Collaborative group study of the epidemiology of acute lymphoblastic leukaemia subtypes: Background and first report. *Leuk. Res.*, **9**, 715-733.
GREAVES MF. (1988). Speculations on the cause of childhood acute lymphoblastic leukaemia. *Leukemia*, **2**, 120-125.
GREAVES MF AND CHAN LC. (1986). Annotation: Is spontaneous mutation the major 'cause' of childhood acute lymphoblastic leukaemia? *Br. J. Haematol.*, **64**, 1-13.
GUNZ FW. (1966). Studies on the incidence and aetiology of leukaemia in New Zealand. *N. Z. Med. J., Haematology Supplement*. **65**, 857-862.
HEWITT D. (1955). Some features of leukaemia mortality. *Br. J. Prev. Soc. Med.*, **9**, 81-88.
KINLEN L. (1988). Evidence for an infective cause of childhood leukaemia: Comparison of a Scottish new town with nuclear reprocessing sites in Britain. *Lancet*, **2**, 1323-1327.
KLEBANOFF MA, READ JS, MILLS JL AND SHIONO PH. (1993). The risk of childhood cancer after neonatal exposure to vitamin K. *N. Engl. J. Med.*, **329**, 905-908.
MANTEL N. (1963). Chi-square tests with one degree of freedom; extensions of the Mantel-Haenszel procedure. *J. Am. Stat. Assoc.*, **58**, 690-700.
MATTHEWS M. (1994). A review of fallout deposition and milk contamination. *Radiat. Protection News Notes*, **26**, 15-17.
MCWHIRTER WR AND PETROESCHEVSKY AL. (1991). Incidence trends in childhood cancer in Queensland, 1973-1988. *Med. J. Aust.*, **154**, 453-455.
MEDICAL STATISTICS BRANCH OF THE DEPARTMENT OF HEALTH. (1955). *Report of the Medical Statistician on Cancer Morbidity and Mortality in New Zealand.* Department of Health: Wellington.
NEGLIA JP AND ROBISON LL. (1988). Epidemiology of the childhood acute leukemias. *Pediatr. Clin. N. Am.*, **35**, 675-692.
NEW ZEALAND HEALTH INFORMATION SERVICE. (1995). *Cancer: New Registrations and Deaths 1992.* Ministry of Health: Wellington.
OLSEN JH, HERTZ H, BLINKENBERG K AND VERDER H. (1994). Vitamin K regimens and incidence of childhood cancer in Denmark. *Br. Med. J.*, **308**, 895-896.
PARKIN DM, STILLER CA, DRAPER GJ, BIEBER CA, TERRACINI B AND YOUNG JL. (1988*a*). *International Incidence of Childhood Cancer*. International Agency For Research on Cancer: Lyon.
PARKIN DM, STILLER CA, DRAPER GJ AND BIEBER CA. (1988b). The international incidence of childhood cancer. *Int. J. Cancer*, **42**, 511-520.
POLEDNAK AP. (1986). Recent trends in incidence and mortality rates for leukemias, and in survival rates for childhood acute lymphocytic leukemia, in Upstate New York. *Cancer*, **57**, 1850-1858.
PUBLIC HEALTH COMMISSION OF NEW ZEALAND. (1993). *Our Health, Our Future. The State of The Public Health in New Zealand 1993.* Public Health Commission: Wellington.
PUBLIC HEALTH COMMISSION OF NEW ZEALAND. (1994). *Our Health, Our Future. The State of the Public Health in New Zealand 1994.* Public Health Commission: Wellington.
REVIEW COMMITTEE ON ETHNIC STATISTICS. (1988). *Report of the Review Committee on Ethnic Statistics*. pp.1-132. Department of Statistics: Wellington.
ROSS JA, DAVIES SM, POTTER JD AND ROBISON LL. (1994). Epidemiology of childhood leukemia, with a focus on infants. *Epidemiol. Rev.*, **16**, 243-272.
SMITH AH AND PEARCE NE. (1984). Determinants of differences in mortality between New Zealand Maoris and non-Maoris aged 15-64. *N. Z. Med. J.*, **97**, 101-108.
STATISTICS NEW ZEALAND. (1995). *Demographic Trends 1994.* Statistics New Zealand: Wellington.
STEWART A AND KNEALE GW. (1969). Role of local infections in the recognition of haemopoietic neoplasms. *Nature*, **223**, 741-742.
VAN HOFF J, SCHYMURA MJ AND MCCREA CURNEN MG. (1988). Trends in the incidence of childhood and adolescent cancer in Connecticut, 1935-1979. *Med. Pediatr. Oncol.*, **16**, 78-87.
WATERHOUSE J, MUIR C, CORREA P, POWELL J AND DAVIS W. (1976). *Cancer Incidence in Five Continents.* Vol III. International Agency for Research on Cancer: Lyon.